# Heterostructural Graphene Quantum Dot/MnO_2_ Nanosheets toward High‐Potential Window Electrodes for High‐Performance Supercapacitors

**DOI:** 10.1002/advs.201700887

**Published:** 2018-03-06

**Authors:** Henan Jia, Yifei Cai, Jinghuang Lin, Haoyan Liang, Junlei Qi, Jian Cao, Jicai Feng, WeiDong Fei

**Affiliations:** ^1^ State Key Laboratory of Advanced Welding and Joining Harbin Institute of Technology Harbin 150001 China

**Keywords:** aqueous supercapacitor, GQDs, heterostructures, MnO_2_, ultrahigh potential window

## Abstract

The potential window of aqueous supercapacitors is limited by the theoretical value (≈1.23 V) and is usually lower than ≈1 V, which hinders further improvements for energy density. Here, a simple and scalable method is developed to fabricate unique graphene quantum dot (GQD)/MnO_2_ heterostructural electrodes to extend the potential window to 0–1.3 V for high‐performance aqueous supercapacitor. The GQD/MnO_2_ heterostructural electrode is fabricated by GQDs in situ formed on the surface of MnO_2_ nanosheet arrays with good interface bonding by the formation of Mn—O—C bonds. Further, it is interesting to find that the potential window can be extended to 1.3 V by a potential drop in the built‐in electric field of the GQD/MnO_2_ heterostructural region. Additionally, the specific capacitance up to 1170 F g^−1^ at a scan rate of 5 mV s^−1^ (1094 F g^−1^ at 0–1 V) and cycle performance (92.7%@10 000 cycles) between 0 and 1.3 V are observed. A 2.3 V aqueous GQD/MnO_2_‐3//nitrogen‐doped graphene ASC is assembled, which exhibits the high energy density of 118 Wh kg^−1^ at the power density of 923 W kg^−1^. This work opens new opportunities for developing high‐voltage aqueous supercapacitors using in situ formed heterostructures to further increase energy density.

## 1 Introduction

With the tremendous growth in renewable power tools, it is imperative to meet the increasing demand for high‐performance electrochemical energy sources. Supercapacitors which pose faster charge/discharge rates and higher power density are becoming hot topics of current research worldwide.^1^, ^2^, ^3^, ^4^ However, the practical application of supercapacitors has long been a challenge because of their limited energy density. Thus, a great deal of research effort has been devoted on high energy density supercapacitors. According to the equation of energy density *E* = 1/2 *CV* ^2^, the energy density (*E*) of supercapacitors can be enhanced by increasing either voltage window (*V*) or specific capacitance (*C*).^5^

For high specific capacitance, one of the research efforts should concentrate on using transition metal oxides (TMO) (e.g., MnO_2_, RuO_2_) as electrode materials.^6^, ^7^ They can provide great specific capacitance due to the pseudocapacitive characteristic.^8^, ^9^ Among TMO materials, MnO_2_ is one of the most potential materials for supercapacitors due to its high theoretical specific capacitance, environmental friendliness, and the high practical voltage window (about 1 V).^2^ However, it has suffered from intrinsically low conductivity and specific surface area, which severely restrict its further development in practical application of supercapacitors. In this regard, integrating nanostructured MnO_2_ and conductive carbon materials to fabricate novel hybrid nanostructures is a plausible solution to overcome this obstacle.^4^ Further, some research efforts have been accordingly performed to synthesize hybrid nanostructures electroactive materials for constructing supercapacitors with considerable performance. Some researchers accordingly synthesized high‐performance nanostructured MnO_2_‐carbon materials electrodes, whose specific capacitance is close to theoretical value. However, the undesirable contact resistances that produced by the weak and noncoherent TMO/conductor interfaces lead to sluggish kinetics for charge transport, which requires further improvement.^10^, ^11^

In order to further improve the energy density, some researchers have concentrated on enlarging the voltage window of supercapacitors. The applications of aqueous electrolytes have been limited by their theoretical voltage window (≈1.23 V) and the practical voltage window is mostly lower than about 1 V for supercapacitors.^12^ To extend the voltage window, various techniques have been applied, such as dual shuttle‐ion electrolytes,^13^, ^14^ pH adjustment of electrolytes,^15^ and concentrated electrolytes.^16^, ^17^, ^18^ All of these methods have complex processing technologies and sacrifice capacitance, so being difficult for practical applications. Recently, Zhu and co‐workers focused on the voltage undertaken by structural modification through Na^+^ deintercalation and intercalation and found that the potential of the Mn_3_O_4_ can be extended to 1.3 V through this method. Although the specific capacitance of Mn_3_O_4_ is 366 F g^−1^, it still remarkably exhibits a large energy density of up to 86 Wh kg^−1^.^19^ Undoubtedly, the potential of one electrode is effectively undertaken by the design of the material structure, which is the best and most practical way to expand the working voltage. Jiang and co‐workers and Mertin et al. used first‐principle calculations to speculate that conductive materials can form heterostructures with TMO through covalent bond, which has unique voltage drop in semiconductive region.^20^, ^21^ These works provide a novel design idea in which the voltage undertaken between conductive carbon materials and TMO has potential to undertake some voltage from external electric fields to broaden the potential of electrodes. Furthermore, more efforts may be devoted to further exploring better electrodes which enlarge the potential without sacrificing its specific capacitance.

Graphene quantum dots (GQDs), with size typically among several tens of nanometers,^22^ have attracted extensive attention due to their unique conductivity, quantum confinement, and the control of bandgap.^23^ Choi and co‐workers regulated the bandgap of GQDs to form GQDs/ZnO heterostructures through the creation of Zn—O—C bonds, which revealed superior optical characteristics and unique blue emissions that have not been seen in pure ZnO.^24^ Moreover, some studies have reported that GQDs can improve the poor conductivity of TMO, which is beneficial for further obtaining superior specific capacitance.^7^, ^25^ Undoubtedly, considerable attention should be devoted into exploring GQDs‐based heterostructural electrodes, which have superior specific capacitance and expanded potential window of electrodes for high‐performance supercapacitors.

Based on the above discussion, we report the design and development of in situ fabricated GQDs/MnO_2_ heterostructural electrodes for high‐performance supercapacitors. GQDs/MnO_2_ heterostructural electrodes include in situ formed GQDs on the surface of MnO_2_ nanosheet arrays through the formation of Mn—O—C covalent bond by plasma enhanced chemical vapor deposition (PECVD). The GQDs/MnO_2_ heterostructural electrodes not only enlarged the operating potential window of the supercapacitor from 0–1 to 0–1.3 V through voltage drop in heterostructural region but also improved the capacitive performance to reach superior specific capacitance (1170 F g^−1^) in 0–1.3 V. Furthermore, we assembled GQDs/MnO_2_ heterostructural electrodes as positive electrode and nitrogen‐doped graphene (NG) as negative electrodes to construct an asymmetric supercapacitor (ASC). The fabricated 2.3 V aqueous ASC exhibits the ultrahigh energy density of 118 Wh kg^−1^ at the power density of 923 W kg^−1^ with excellent rate performance. This work demonstrates the possibility of extending voltage window and improving specific capacitance in aqueous electrolytes to increase the energy density that can meet the needs of practical application.

## 2 Results and Discussions

**Figure 1:**
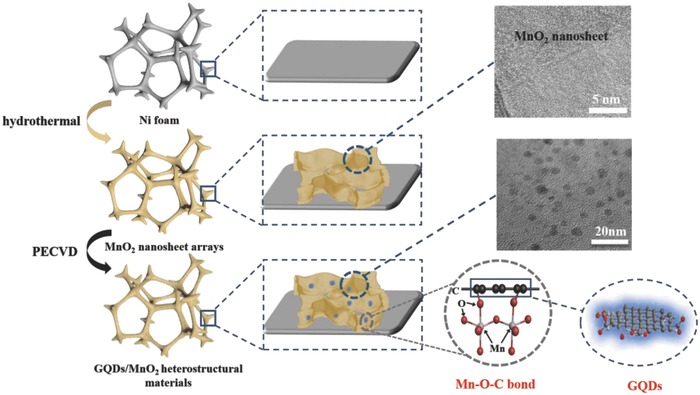
Fabrication process of GQD/MnO_2_ heterostructural materials.

In this paper, the GQDs/MnO_2_ heterostructural materials on Ni foam substrate were synthesized through a facile and effective method. **Figure** **1** illustrates the two‐step synthesis of GQDs/MnO_2_ heterostructural electrodes using hydrothermal and PECVD synthesis. In the first step, a facile hydrothermal method followed by vacuum drying made MnO_2_ nanosheet arrays grown vertically on clean Ni foam substrate (step 1). In the second step, the PECVD process resulted in the in situ formation of GQDs on the surface of MnO_2_ nanosheets (step 2). In this step, we creatively use CO_2_ to replace common hydrocarbons (such as CH_4_) as carbon source to form GQDs/MnO_2_ heterostructures at low temperatures (350 °C). In our previous report,^26^, ^27^ MnO_2_ was synthesized through the same hydrothermal process reported in this paper, and we chose CH_4_ as carbon source in PECVD process. However, the results show that there is no GQDs formation, and we fabricated nanosized core–shell graphene–MnO_2_ nanosheet arrays. The pure MnO_2_ nanosheets are transformed into core–shell graphene–MnO_2_ nanoparticles by using CH_4_, which reveals that introducing CH_4_ in PECVD is beneficial for the growth of graphene. Moreover, Chen and co‐workers reported that H_2_ gas, which decomposes from CH_4_, can change the morphology of manganese oxide.^28^ Lu and co‐workers reported that hydrogen, which can decompose from common hydrocarbons, can partly reduce MnO_2_ nanosheets.^29^ This may cause the phase instability of pure MnO_2_ nanosheets. Furthermore, as previously reported, H_2_ can promote the subsequent nucleation and growth of graphene without GQDs.^30^ Therefore, in order to realize the GQDs growth in PECVD process, we creatively use CO_2_ (does not contain hydrogen atoms) as the carbon source to replace common hydrocarbons, and the obtained samples labeled as GQDs/MnO_2_‐X, where X means deposition time (1–10 min). Specific details and the calculation of mass loading are provided in the Experimental Section (Supporting Information).

Thermogravimetric analyses (TGA) were employed to determine the actual content of each component in all GQDs/MnO_2_ samples, as shown in Figure S1 (Supporting Information). The experiments were performed up to 700 °C in air at a heating rate of 5 °C min^−1^. Under these conditions, the GQDs are burned up while MnO_2_ turned into Mn_2_O_3_.^31^, ^32^ Figure S1 (Supporting Information) provides representative TGA curves of MnO_2_ and all GQDs/MnO_2_ samples. Typically, the weight loss values of MnO_2_, GQDs/MnO_2_‐1, GQDs/MnO_2_‐3, GQDs/MnO_2_‐5, and GQDs/MnO_2_‐10 are found to be 7.77, 8.27, 9.77, 10.54, and 11.29 wt%, respectively. Accordingly, the mass ratio of MnO_2_/GQDs for GQDs/MnO_2_‐1, GQDs/MnO_2_‐3, GQDs/MnO_2_‐5, and GQDs/MnO_2_‐1 can be derived to be 61.1/1, 30.1/1, 23.7/1, and 19.6/1, respectively. The mass percentage of GQDs for GQDs/MnO_2_‐1, GQDs/MnO_2_‐3, GQDs/MnO_2_‐5, and GQDs/MnO_2_‐10 can be derived to be 1.61, 3.22, 4.04, and 4.85 wt%, respectively, as shown in Table S1 (Supporting Information).

**Figure 2:**
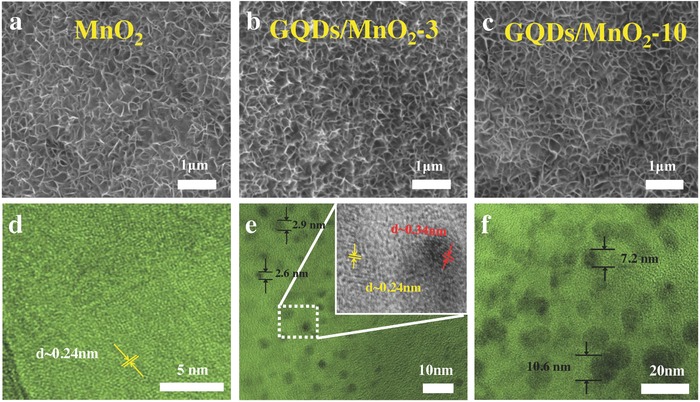
SEM images of a) MnO_2_ nanosheet arrays, b) GQDs/MnO_2_‐3, and c) GQDs/MnO_2_‐10. TEM images of d) MnO_2_ nanosheet arrays, e) GQDs/MnO_2_‐3, and f) GQDs/MnO_2_‐10.

The scanning electron microscope (SEM) and transmission electron microscope (TEM) images of pure MnO_2_ nanosheet arrays and GQDs/MnO_2_ heterostructural materials with different PECVD deposition time are shown in **Figure** **2** and Figure S2 (Supporting Information). As shown in Figure 2a, numerous wrinkled MnO_2_ nanosheets are vertically and densely growing on the substrate. All the nanosheets are interconnected with each other to form a network‐like structure, aiming to facilitate the fast transport of electrons in supercapacitors. Figure 2d shows the high‐resolution TEM (HRTEM) image of MnO_2_ nanosheet arrays scratched from substrate. It demonstrates that the MnO_2_ nanosheet is ultrathin with the polycrystalline structure, in which the lattice spacing of lattice fringes was found to be 0.24 nm, corresponding to the (−111) plane of MnO_2_.^6^

The SEM images of GQDs/MnO_2_ heterostructural materials with different PECVD deposition time are shown in Figure 2b,c and Figure S2a,b (Supporting Information). The nanosheet structure remains in GQDs/MnO_2_ heterostructural materials with different deposition times, which is vertical and highly porous. These results suggest that the deposition of GQDs on the surface of MnO_2_ nanosheets by PECVD process caused no change in the nanosheet morphology. Figure 2e exhibits HRTEM images of the GQDs/MnO_2_‐3. Many dots are uniformly distributing in the field of vision, indicating that a large number of GQDs are in situ formed on the surfaces of the MnO_2_ sheets through PECVD process. The sizes of GQDs in GQDs/MnO_2_‐3 are ≈2–3 nm, and the in situ formation of GQDs onto the surface of MnO_2_ nanosheets is possibly ascribed to the strong chemical interactions between GQDs and polycrystalline MnO_2_ nanosheets (discussed in X‐ray photoelectron spectroscopy (XPS) analysis). As shown in the inset of Figure 2e, the lattice spacing of 0.34 nm is excellently indexed to the (002) spacing of graphitic carbon.^7^ Furthermore, the size of GQDs can be continuously changed from 1 to 2 nm (GQDs/MnO_2_‐1, see Figure S2a, Supporting Information) to ≈10 nm (GQDs/MnO_2_‐10, see Figure 2f), which means that the size of GQDs can be adjusted through different technology parameters. Because the size of GQDs is closely related to the bandgap structure, GQDs with different size may have different effects on electrochemical performance. Compared with conventional CQDs, GQDs are generally smaller and of higher crystallinity.^22^ And GQDs have larger specific surface areas and more accessible edges, which results in an influence of capacitance. In our case, HRTEM images of the GQDs/MnO_2_‐3 show that many dots are uniformly distributed in the field of vision, as shown in Figure S3a (Supporting Information). The sizes of GQDs in GQDs/MnO_2_‐3 are ≈2–3 nm, and the lattice spacing of 0.34 nm is excellently indexed to the (002) spacing of graphitic carbon, which implies small sizes and high crystallinity, and this agrees with the previous report about GQDs.^23^, ^24^

**Figure 3:**
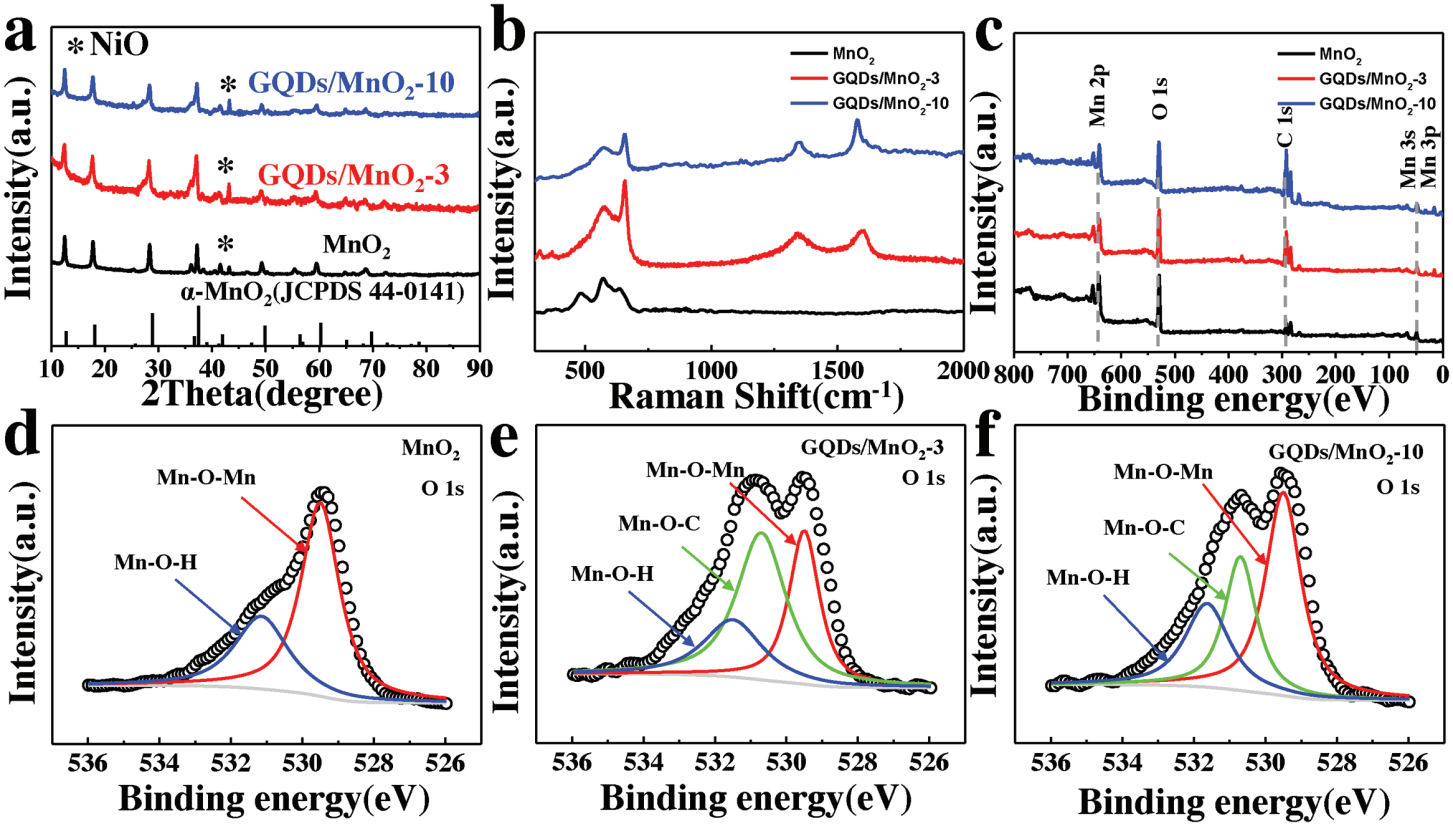
a) The representative XRD patterns for as‐prepared MnO_2_, GQDs/MnO_2_‐3, and GQDs/MnO_2_‐10. b) Raman spectra for MnO_2_, GQDs/MnO_2_‐3, and GQDs/MnO_2_‐10. c) Survey XPS spectrum for MnO_2_, GQDs/MnO_2_‐3, and GQDs/MnO_2_‐10. High‐resolution O 1s XPS spectra for d) MnO_2_, e) GQDs/MnO_2_‐3, and f) GQDs/MnO_2_‐10.

The X‐ray powder diffraction (XRD) is investigated to help understand the crystal structure of MnO_2_ nanosheet arrays before and after in situ formed GQDs by PECVD. The results in **Figure** **3**a show the crystal structures of MnO_2_ nanosheet arrays, GQDs/MnO_2_‐3, and GQDs/MnO_2_‐10. All of the samples were scratched away from the Ni foam to remove the influence. The diffraction peaks of all samples agree with the XRD patterns of α‐MnO_2_ (JCPDS No. 44‐0141).^33^ And there is one additional small peak located at 43.1° in addition to MnO_2_, which means the existence of impurities. Based on previously reported results, the XRD diffraction peak at 43.3° can be indexed to (200) of NiO,^34^, ^35^ which probably comes from the gradual oxidation of Ni foam in hydrothermal and cannot be avoided when scratching active materials from the Ni foam for XRD measurement. Moreover, carbon materials peaks were not observed in XRD after PECVD deposition indicated the ultralow mass loading of GQDs on MnO_2_ nanosheets.

Raman spectroscopy has become one of the most sensitive methods to analyze carbon materials. Thus, the Raman spectrum was investigated for MnO_2_ nanosheet arrays before and after in situ formed GQDs. The Raman spectra of MnO_2_ nanosheets, GQDs/MnO_2_‐3, and GQDs/MnO_2_‐10 are shown in Figure 3b. The weak peaks in the range of 501–640 cm^−1^ come from MnO_2_ nanosheets, in agreement with the previous report about α‐MnO_2_.^33^ After the GQDs were in situ formed on the surface of MnO_2_ nanosheets, the Raman spectrum also contained characteristic D and G peaks of carbon materials at ≈1350 and 1580 cm^−1^, which implies the existence of carbon materials. Combined with the TEM results, this demonstrates that the carbon materials are GQDs. Moreover, as shown in Figure S3b (Supporting Information), an additional peak located in 2695 cm^−1^, corresponding to 2D band, can also be observed. This weak 2D band without a typical graphite shoulder shows low *I*_2D_/*I*_G_ ratio (0.5) implying the few‐layered feature of GQDs in GQDs/MnO_2_,^25^ which further prove the formation of GQDs.^22^ The detailed discussion is shown in Figure S3 (Supporting Information). The peak intensity ratio between the disorder‐induced D peak and sp^2^ vibration G peak (*I*_D_/*I*_G_) generally provides an effective indicator for comparing the degree of crystallinity of carbon materials. The *I*_D_/*I*_G_ ratios of GQDs/MnO_2_‐3 and GQDs/MnO_2_‐10 are 0.98 and 0.8, respectively. This indicates that the disordered structure and edge planes of GQDs are decreased after prolonging the deposition time of PECVD, which is mainly attributed to the edges of GQDs with a low stability that are etched away through growth of GQDs and finally become regular and straighter.

The XPS spectra of MnO_2_ nanosheet arrays before and after in situ formed GQDs by PECVD are shown in Figure 3c. The survey scan spectrum reveals the existence of C, Mn, and O elements in all samples. GQDs/MnO_2_‐3 and GQDs/MnO_2_‐10 exhibit newly stronger peak of C 1s than MnO_2_ nanosheet arrays, corresponding to that the GQDs are in situ synthesized on the surface of MnO_2_ nanosheet arrays. Figure S4 (Supporting Information) shows the Mn 2p core‐level spectra of MnO_2_ nanosheets, GQDs/MnO_2_‐3, and GQDs/MnO_2_‐10, and all curves consist of two peaks, which correspond to the spin–orbit doublet of Mn 2p^3/2^ and Mn 2p^1/2^, respectively. The Mn 2p^3/2^ peak of all samples can be deconvoluted into three peaks as Mn^4+^ (642.8 eV), Mn^3+^ (641.8 eV), and Mn^2+^ (640.7 eV).^1^, ^36^, ^37^ The proportion of Mn^4+^ peaks shows almost no change after PECVD process, demonstrating the phase stability in the PECVD process.^38^ Moreover, chemical titration was used to further quantitatively confirm the average valence for Mn in different samples.^39^ The composition for MnO_2_ nanosheets, GQDs/MnO_2_‐3, and GQDs/MnO_2_‐10 was determined to be MnO_1.76_, MnO_1.74_, and MnO_1.71_, respectively. The detailed high‐resolution spectra of O 1s of all samples are shown in Figure 3d–f and Figure S2c,f (Supporting Information). Further, all O 1s can be divided into several peaks. The peak around 529.5 and 531.1 eV corresponds well with Mn—O—Mn and Mn—O—H bonds.^40^ The Mn—O—H bond could originate from the structural water. Apart from the Mn—O—Mn and Mn—O—H bonds, an unusual peak around 530.7 eV confirms the formation of Mn—O—C bonds in all GQDs/MnO_2_ heterostructural materials.^41^ Based on this, we can infer that the heterostructural materials were formed as the result of π–π stacking between the GQDs and MnO_2_ nanosheets.^42^

The tuning of PECVD deposition time plays an important role in the formation of Mn—O—C bonds. As shown in **Table** **1**, the contents of Mn—O—C change significantly in different time. By prolonging the deposition time to 5 min, the Mn—O—C contents show a gradual increase, from 0 to 55.1%, which means the increasing formation of Mn—O—C bonds. Furthermore, a gradual decrease to 28% through prolonging the deposition time to 10 min suggests the time‐dependent structural evolution of GQDs on MnO_2_ nanosheets. The formation mechanism of GQDs can be deduced as follows. Briefly, the fresh GQDs nucleuses are first in situ generated on MnO_2_ nanosheets through Mn—O—C bonds. Then, the growing GQDs monomers transform to ≈5 nm nanosheet structure to reduce surface free energy through Mn—O—C bonds. With extended deposition process, the exterior clustered GQDs nucleus continues to directly grow up at the edge of GQDs without the formation of Mn—O—C bonds. Therefore, the content of C increases and the content of Mn—O—C decreases with further prolonging the deposition time to 10 min. Furthermore, the contents of Mn—O—Mn and Mn—O—H also indicate that some of the Mn—O—Mn and Mn—O—H have been transformed into Mn—O—C. The interfacial interaction of the Mn—O—C bond between GQDs and MnO_2_ should be beneficial for charge transfer and structure stability, and the formation of covalent bond in the interface with good interfacial bonding will benefit the formation of reliable heterostructures. Therefore, the GQDs/MnO_2_ heterostructural materials may be ideal materials for high‐performance supercapacitor electrodes.

**Table 1:** The contents of Mn—O—Mn, Mn—O—H, and Mn—O—C in all samples

	Samples				
	MnO_2_	GQDs/MnO_2_‐1	GQDs/MnO_2_‐3	GQDs/MnO_2_‐5	GQDs/MnO_2_‐10
Mn—O—Mn	63.6%	64.1%	30.8%	23.7%	47.7%
Mn—O—H	36.4%	16.4%	21.8%	21.2%	24.3%
Mn—O—C	0%	19.5%	47.4%	55.1%	28%

**Figure 4:**
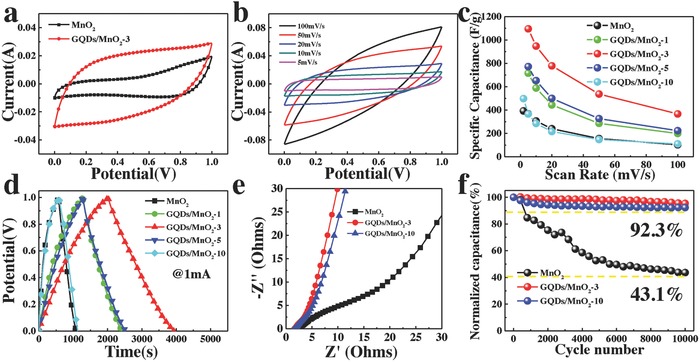
CV curves of MnO_2_ and GQDs/MnO_2_‐3 at a scan rate of 20 mV s^−1^. b) CV curves of GQDs/MnO_2_‐3 at different scan rates (5–100 mV s^−1^). c) Specific capacitances of all samples as a function of the scan rate. d) GCD curves of all samples at current of 1 mA. e) Nyquist plots in a frequency range from 0.01 Hz to 100 kHz for all samples. f) Cycling stability tests over 10 000 cycles for MnO_2_, GQDs/MnO_2_‐3, and GQDs/MnO_2_‐10.

Next, the capacitance behaviors of the MnO_2_ nanosheets and GQD/MnO_2_ heterostructural materials were investigated to compare the capacitance contribution of GQDs. The cyclic voltammetry (CV) curves of all samples with various sweep rates ranging from 5 to 100 mV s^−1^ in the potential range of 0–1 V are shown in **Figure** **4**b and Figure S5a–d (Supporting Information). It can be seen that the CV curves area of GQD/MnO_2_‐3 is the largest, which means that GQD/MnO_2_‐3 has the highest specific capacitance (Figure 4b). Figure 4a shows the CV curves of MnO_2_ nanosheet arrays and GQDs/MnO_2_‐3 measured at 20 mV s^−1^. Compared with that of pure MnO_2_ nanosheet arrays, the CV curves of GQDs/MnO_2_‐3 are relatively rectangular in shape and exhibit a larger area. These indicate ideal pseudocapacitive characteristics and higher capacitance than that of pure MnO_2_ nanosheet arrays. Furthermore, all CV curves of GQDs/MnO_2_ heterostructural electrodes at the low rate still maintain the “rectangle‐shape,” which exhibits that the electron transfer in the GQDs/MnO_2_ heterostructural electrodes is fairly rapid. The specific capacitances of all samples, which were calculated by integrating the voltammetric charge in the CV curves, are plotted in Figure 4c. At the same scan rate, the consequent specific capacitance for GQD/MnO_2_‐3 was much higher than that of others. The crest specific capacitance of 1094 F g^−1^ at a scan rate of 5 mV s^−1^ can be achieved with GQD/MnO_2_‐3, which is only 393 F g^−1^ of pristine MnO_2_ nanosheet arrays. Further prolonging the deposition time, the specific capacitance would follow a downward trend. Obviously, in situ forming GQDs on MnO_2_ nanosheets can improve the specific capacitance, because of the abundant edge sites provided by small‐size GQDs and the good conductivity correlated to Mn—O—C bond.^43^ However, forming large‐size GQDs on MnO_2_ nanosheets has a negative effect on electrode performance, primarily because of the sacrifice of contact area between MnO_2_ and electrolyte. Figure 4d displays the comparison of galvanostatic charge–discharge (GCD) curves for all as‐prepared electrodes at 1 mA. The same trend of capacitance can also be calculated by comparison of discharging time of various samples at the same charge/discharge current. As shown in Figure S5e (Supporting Information), the *iR* drop of the GQD/MnO_2_‐3 electrode is much smaller than that of the MnO_2_ nanosheet arrays, implying the fast diffusion of ions and a low equivalent series resistance after the introduction of GQDs.^44^ Undoubtedly, these results convincingly illustrate that in situ formed GQDs can improve the conductivity and electrochemical performances of MnO_2_ nanosheet arrays.

Electrochemical impedance spectroscopy (EIS) was conducted to understand the ion diffusion and conductivity behavior at a frequency range of 100 kHz to 0.01 Hz. The Nyquist plots for all samples are shown in Figure 4e. All the electrodes present a similar shape in the three regions. The vertical line at low frequency indicates the diffusive behavior of electrolyte ions in the electrode,^8^ and the more vertical line of GQD/MnO_2_‐3 than others suggests lower ion diffusion resistance and faster ion diffusion behavior.^45^ The slope of ≈45° lines reveals the diffusion behavior on the electrolyte/electrode interface.^46^ In the high‐frequency region, the diameter of semicircle denotes charge transfer resistance (*R*_ct_).^47^ According to the equivalent circuit, the decreasing of *R*_ct_ from MnO_2_ nanosheet arrays to the GQDs/MnO_2_ heterostructural electrodes (from 2.5 to 1.45 Ω) should be associated with the in situ formed GQDs on the surface of MnO_2_ nanosheets that is in accordance with the results of *iR* drop. Based on above TGA, the mass percentage of GQDs is increased with prolonging the PECVD process time. And the mass percentage of GQDs for GQDs/MnO_2_‐10 is 4.85 wt%, which is larger than GQDs/MnO_2_‐3 (3.22 wt%). The EIS data thus demonstrate that increased mass percentage of GQDs presents enhanced conductivity in both the serial and charge transfer parts.

Long‐time cycling performance is also an important requirement for supercapacitors, the cycling performance of these samples was examined by GCD measurements, as shown in Figure 4f and Figure S5f (Supporting Information). For MnO_2_ nanosheet arrays, the specific capacitance decreased to 43.1% of the initial capacitance after 10 000 cycles. For GQDs/MnO_2_‐3, the specific capacitance retained 95.4% of the initial value after continuous 10 000 cycles. The superior stability is ascribed to the unique morphology and interface structure: GQDs can prevent the aggregation of MnO_2_ nanosheets to keep electrode structure stable resulting in enhanced cycling life, as shown in Figure S6 (Supporting Information). The morphology and structure of MnO_2_ nanosheets suffer from serious agglomeration and collapse, which are marked by arrows in Figure S6a (Supporting Information). Nevertheless, the morphology and structure of GQDs/MnO_2_‐3 and GQDs/MnO_2_‐10 remain unchanged in comparison with MnO_2_ nanosheets, as shown in Figure S6b,c (Supporting Information). The poor cycling stability of MnO_2_ nanosheets can be contributed to the structural breakdown, electrode pulverization, and poor conductivity during long‐term cycling. The existence of Mn—O—C covalent bond in the interface can suppress the strain and stress that pulverize electrodes. The XPS measurements were performed to investigate the average oxidation state of Mn in MnO_2_ for pure MnO_2_ nanosheets and GQDs/MnO_2_ electrodes after cycling. Figure S7a–c (Supporting Information) shows the Mn 2p core‐level spectra of MnO_2_ nanosheets, GQDs/MnO_2_‐3, and GQDs/MnO_2_‐10 after cycling test, and all curves consist of Mn 2p^3/2^ and Mn 2p^1/2^, respectively. Compared with before cycling test, the Mn^4+^ peaks of GQDs/MnO_2_ samples show almost no change after cycling test, demonstrating that oxidized Mn^4+^ was basically unchanged. However, the Mn 2p core‐level spectra of pure MnO_2_ nanosheets show significant change after cycling, as shown in Figure S7a (Supporting Information). The proportions of Mn^4+^ peaks of pure MnO_2_ decrease after cycling test, indicating that the Mn^4+^ is reduced during the electrochemical reactions.^39^ And the composition for MnO_2_, GQDs/MnO_2_‐3, and GQDs/MnO_2_‐10 after cycling was determined to be MnO_1.53_, MnO_1.67_, and MnO_1.64_, respectively.

For comparison, we fabricated GQDs through a facile solution method and further decorated with MnO_2_ nanosheet arrays by hydrothermal method (details in the Experimental Section), labeled H‐GQDs@MnO_2_. As shown in Figure S8 (Supporting Information), the specific capacitance of H‐GQDs@MnO_2_ can reach 550 F g^−1^ at a scan rate of 5 mV s^−1^, which means that GQDs play an important role to improve the conductivity and electrochemical performance. However, there is a limited improvement of specific capacitance and a poor cycling stability (62.2% retention after 5000 cycles), which can be attributed to the inexistence of Mn—O—C bonds through hydrothermal method. As a result, it suggests that Mn—O—C bonds contribute to the cycling performance. The good cycling performance, together with the high specific capacitance discussed in the above, implies a good potential material for the present high energy/power density supercapacitors.

**Figure 5:**
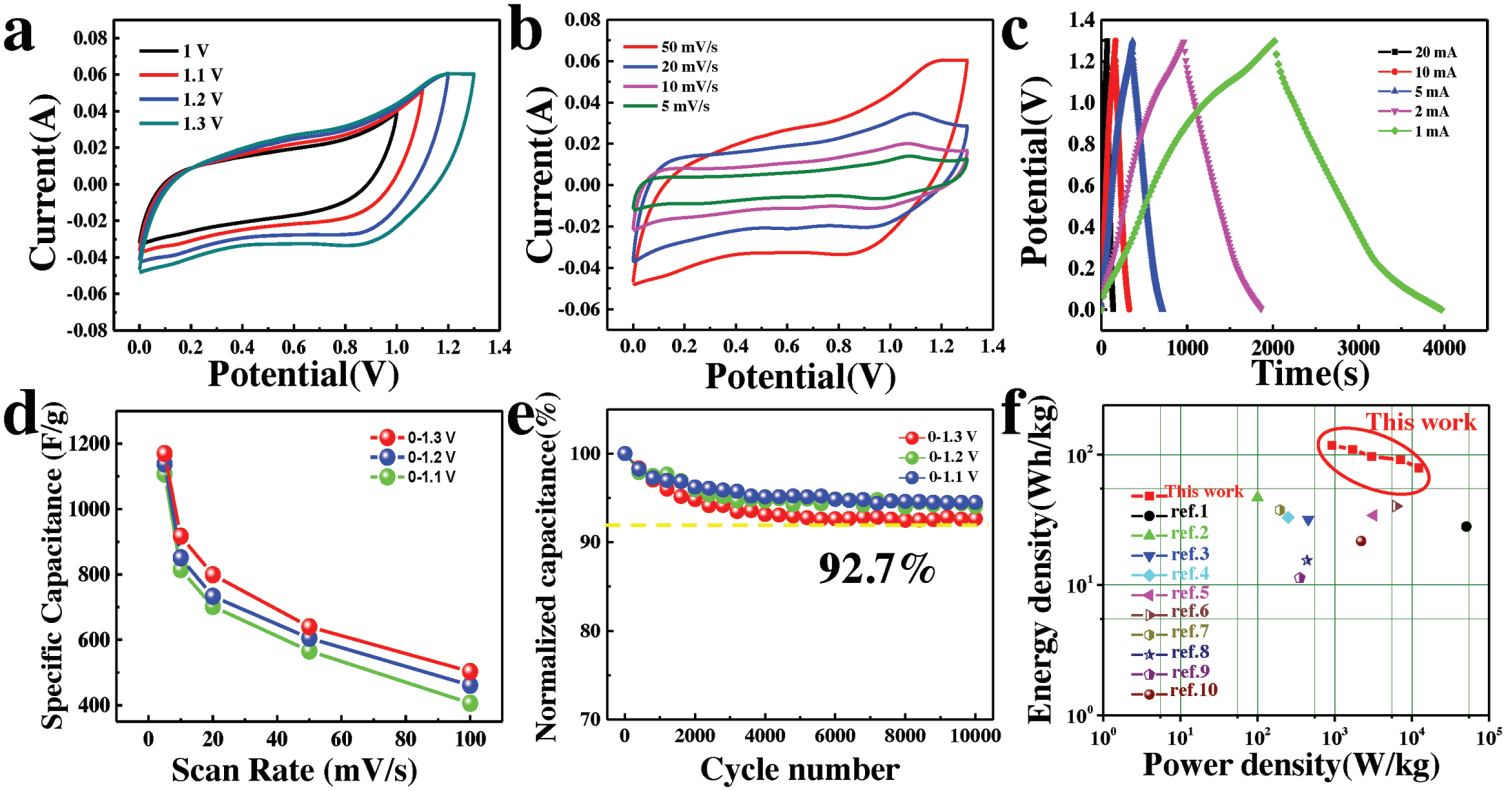
CV curves of GQDs/MnO_2_‐3 in different potential windows of 0–1, 0–1.1, 0–1.2, and 0–1.3 V. b) CV curves of GQDs/MnO_2_‐3 at different scan rates (5–50 mV s^−1^). c) GCD curves of the GQDs/MnO_2_‐3 in 0–1.3 V at different current. d) The specific capacitances of the GQDs/MnO_2_‐3 as a function of scan rate in different potential windows. e) Cycling stability of the GQDs/MnO_2_‐3 in different potential windows. f) The Ragone plots of the GQDs/MnO_2_‐3//NG ASC and reported MnO_2_‐based ASCs in literature.

To evaluate the electrochemical behavior of the unique GQDs/MnO_2_‐3 heterostructures, typical CV curves were recorded at a scan rate of 50 mV s^−1^ in different potential windows of 0–1.0, 0–1.1, 0–1.2, and 0–1.3 V, respectively, as shown in **Figure** **5**a. A slight deformation of CV curves occurred at a potential window of beyond 1.4 V, indicating the occurrence of some irreversible reactions. This suggests that the potential window of GQDs/MnO_2_‐3 electrode can be extended to 1.3 V when using GQDs to form heterostructures. Figure S9 (Supporting Information) shows the digital images of electrolytic cell in different measurement conditions for two typical samples. In the potential window of 1.3 V, the counter electrode (Pt foil) in the electrolytic cell of MnO_2_ nanosheet arrays produces a small amount of bubbles. In contrast, no obvious change of Pt foil can be observed in the electrolytic cell with GQDs/MnO_2_‐3 electrode, suggesting the expanded potential window of GQDs/MnO_2_‐3 electrode. This means that the GQDs/MnO_2_ heterostructural electrode can work stably at 0–1.3 V. Further, H‐GQDs@MnO_2_ cannot work at the potential window beyond 1 V, which means that GQDs themselves do not contribute to the extending of potential window and can only improve the specific capacitance of electrodes. This result implies that the heterostructure in GQDs/MnO_2_ is the key to extending the potential window of one electrode.

Figure 5b and Figure S10 (Supporting Information) show the CV curves of GQDs/MnO_2_ electrodes with deposition time 1, 3, and 10 min at the scan rate from 5 to 50 mV s^−1^ in the potential window of 1.3 V. The CV curves still retain rectangular shape even at 0–1.3 V indicating excellent reversibility and rate capability.^48^, ^49^ Furthermore, the performance of GQDs/MnO_2_‐3 electrode was also evaluated by GCD test at different current of 1–20 mA between 0 and 1.3 V, as shown in Figure 5c. All of these charge/discharge curves at different currents exhibit nearly linear slopes and reveal good symmetry, suggesting excellent capacitive behavior for GQDs/MnO_2_‐3. The specific capacitances of the GQDs/MnO_2_‐3 as a function of scan rate in different potential windows are exhibited in Figure 5d. At the scan rate of 5 mV s^−1^, the specific capacitance of GQDs/MnO_2_‐3 electrode can reach 1170 F g^−1^ in 0–1.3 V, which is still larger than 1138 F g^−1^ in 0–1.2 V, 1107 F g^−1^ in 0–1.1 V, and 1094 F g^−1^ 1.0 V, and it is interesting to find that the specific capacitance of GQDs/MnO_2_‐3 electrode increases with the potential window increases from 1 to 1.3 V. This implies that extension of the potential window to 1.3 V does not result in decreased specific capacitance, but rather enhances the possibility for improving the energy density of supercapacitors. In order to further explore the influence on the stability when extending the potential window, the cycling performance of GQDs/MnO_2_‐3 electrode in 0–1.1, 0–1.2, and 0–1.3 V for 10 000 charge/discharge cycles at a current of 10 mA is shown in Figure 5e. The specific capacitance retentions in 0–1.1, 0–1.2, and 0–1.3 V are 94.5, 94, and 92.7% after 10 000 cycles, indicating expansion of the potential window will not cause obvious deterioration of cycling stability. Hence, extension of potential window to 1.3 V of GQDs/MnO_2_‐3 electrode through unique heterostructures between GQDs and MnO_2_ is a practical and potential way in developing high specific capacitance and high voltage supercapacitors, which means new opportunities to develop high energy density supercapacitors.

Generally, the electrochemical behavior was carried out in a three‐electrode electrochemical measurement. However, the fabrication of a two‐electrode hybrid supercapacitor is crucial for real applications, which is aiming at both high energy and power densities. Herein, we have constructed an ASC by using GQDs/MnO_2_‐3 heterostructural materials as positive electrodes and NG as negative electrodes (Figure S11, Supporting Information). The mass ratio of two electrodes was balanced by the following relationship^50^, ^51^, ^52^

(1) $$m_{+}/m_{-}=\left(C_{-}\times \Delta E_{-}\right)/\left(C_{+}\times \Delta E_{+}\right)$$

where *m* (g) is the mass of the electrode materials (anode or cathode), *C* (F/g) is the specific capacitance, and Δ*E* is the potential window. Figure S11a (Supporting Information) shows CV curves of the typical GQDs/MnO_2_‐3 electrodes and NG at the scan rate of 10 mV s^−1^, which illustrates the potential window of NG is −1 to 0 V. Based on above mentioned results, the theoretical voltage window of the ASC can be extended to 2.3 V. As shown in Figure S11b (Supporting Information), the GQDs/MnO_2_‐3//NG ASC works well when the voltage window reaches 2.3 V. The mass specific capacitance of the GQDs/MnO_2_‐3//NG ASC was calculated based on the total mass of the electroactive materials in anode and cathode. The mass loading of GQDs/MnO_2_‐3//NG ASC is 6.5 mg cm^−2^ and the specific capacitance is shown in Figure S11d (Supporting Information). The GQDs/MnO_2_‐3//NG ASC deliver high capacitances from 160.6 F g^−1^ at 5 mV s^−1^ to 124.4 F g^−1^ at 50 mV s^−1^, demonstrating the superior rate performance. The energy density and power density performance of GQDs/MnO_2_‐3//NG are compared using a Ragone plot (Figure 5f). It shows that by taking advantage of the large specific capacitance and high voltage window, the GQDs/MnO_2_‐3//NG has the highest energy density of 118 Wh kg^−1^ at the power density of 923 W kg^−1^, which is substantially larger than previously reported values. Even at the high power density of 12 351 W kg^−1^, the GQDs/MnO_2_‐3//NG ASC can still exhibit a superior energy density of 79 Wh kg^−1^, demonstrating excellent energy density.

**Figure 6:**
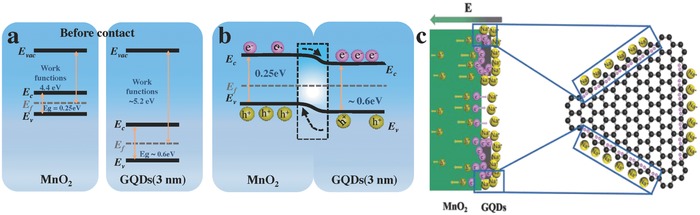
a) Energy diagram of MnO_2_ and GQDs (≈3 nm) before contact. b) Energy diagram of the interface between MnO_2_ and GQDs (≈3 nm) after the formation of a heterojunction. c) The schematic diagram of free electrons accumulating near the GQDs surface.

In order to understand the correlation between the increase in capacitance and the extension of potential window with the heterostructure of GQDs, we propose a schematic energy band diagram, as shown in **Figure** **6**a,b. As previous reports illustrated that the bandgap of GODs increases with the reduction in their size. This is analogous to previous experimental relationship *E*_g_ (eV) = 1.68 eV nm/*L*, where *E*_g_ is energy gap and *L* is size.^53^ Based on our results, the GQDs in GQDs/MnO_2_‐3 are almost 2–3 nm, which means that the bandgap is almost 0.6 eV, and the bandgap of GQDs in GQDs/MnO_2_‐10 (7–10 nm) is almost 0.2 eV. Furthermore, the work function of GQDs is still probably related to their size, the work function of GQDs (≈3 nm) is ≈5.2 eV and that of GQDs (7–10 nm) is 4.5–4.7 eV.^54^, ^55^ In general, at the interface between two materials, a built‐in electric field is established because of their different work functions.^56^, ^57^ The work functions of MnO_2_ and GQDs in GQDs/MnO_2_‐3 are 4.4 and ≈5.2 eV, respectively,^58^ which implies that the free electrons will inject from MnO_2_ to the GQDs at the interface until the Fermi levels are aligned. When MnO_2_ and GQDs contact and form a heterostructure through Mn—O—C covalent bonds, the MnO_2_ is positively charged while GQDs are negatively charged near the interface. Moreover, the built‐in electric field cannot be fully screened due to the small lateral size of GQDs. This enables the free electrons to accumulate near the GQDs surface, as shown in Figure 6c. In addition, the unique GQDs with zigzag edges further cause the free electrons injected from MnO_2_ to accumulate at the edges. Apparently, the free electrons accumulated at the edges of GQDs can induce extra electrostatic attraction with sodium ions, which surely improves the utilization ratio of cations, resulting in a synergistic effect for increasing specific capacitance. However, as for GQDs with the size of almost 7–10 nm, although the Fermi level of MnO_2_ is higher than GQDs, as shown in Figure S12 (Supporting Information), the lower energy bandgap implies that a small number of electrons will inject from MnO_2_ to the GQDs at the interface until the Fermi levels are aligned.^59^ Therefore, although there is the band bending on the MnO_2_ surface, the electron collection efficiency is limited due to a few accumulated electrons. Based on the energy storage mechanism of MnO_2_ 60

(2) $${\mathrm{MnO}}_{2}+M^{+}+e^{-}⇋\mathrm{MnOOM}\left(M^{+}={\mathrm{Li}}^{+},{\mathrm{Na}}^{+},K^{+}\right)$$

the redox activity of the MnO_2_ is limited due to the limited electrons accumulation in GQD/MnO_2_‐10 than that of GQD/MnO_2_‐3, which means the inferior performance than GQDs (≈3 nm). Moreover, GQDs (7–10 nm in GQDs/MnO_2_‐10) have a larger size, which is not beneficial because in this case the ion diffusion into MnO_2_ cannot be guaranteed and the pseudocapacitive reaction cannot be manifested.^61^ The edges of GQDs are always seen as active sites for electrochemical reaction, increasing the size of GQDs may decrease the edge sites of GQDs, which could possess decreased specific capacitance.^62^, ^63^ Therefore, the specific capacitance of GQD/MnO_2_‐10 is largely limited and the same as the pure MnO_2_, which is the result of interaction between the enhancement of conductivity and the inhibition of pseudocapacitance.

Hence, the potential window of our GQDs/MnO_2_ heterostructural materials can be expanded to 1.3 V. Based on abovementioned results, there are two major reasons for the extension of potential window: one is GQDs, and the other is the heterostructures between GQDs and MnO_2_. However, the H‐GQDs@MnO_2_ hybrid electrodes fabricated using hydrothermal method cannot expand the potential window beyond 1 V, which means that the unique heterostructure plays the decisive effect in extending potential window. As previously reported, the formation of covalent bond in the interface with reliable interface bonding will benefit the formation of heterostructures,^24^ indicating that only GQDs and MnO_2_ tightly connected through Mn—O—C bonds can form reliable heterostructures. Furthermore, the valence and conduction bands of GQDs and MnO_2_ bend in vicinity of the interface that can form potential gradient inside in the interface,^21^ meaning that the built‐in electric field of GQDs/MnO_2_ heterostructural region provides potential barrier for electronic transmission. This barrier can undertake partial potential and counteract external electric fields to broaden the potential window for supercapacitors. As the result, we explore new kind of electrodes which enlarge the potential window without sacrifice its specific capacitance for high‐performance and high energy density supercapacitors.

The GQDs/MnO_2_ heterostructural electrodes possess the following advantages for superior electrochemical performance. First, the binder‐free advantages ensure good electrical contact and the vertically aligned nanosheet structure guarantees fast ion/electron transport and sufficient contact with the electrolyte. Moreover, GQDs with superior conductivity and abundant edge sites can facilitate fast reaction kinetics and enhance specific capacitance. Second, the GQDs attached on MnO_2_ nanosheets through Mn—O—C bonds in the interface are beneficial for remitting volumetric expansion and enhancing cycling stability and have the ability to form heterostructures. Third, the unique heterostructures between GQDs and MnO_2_ not only increase the potential window to 1.3 V but also enhance the specific capacitance of the electrode, which can result in the ultrahigh energy density for ASCs. All the aforementioned merits generate the remarkable electrochemical performances of the GQDs/MnO_2_ heterostructural electrodes.

## 3 Conclusions

In summary, a simple strategy has been demonstrated for fabricating GQDs/MnO_2_ heterostructural electrodes for ultrahigh energy density 1.3 V aqueous supercapacitors. The reliable heterostructures between GQDs and MnO_2_ nanosheet were in situ formed with good interface bonding by the formation of Mn—O—C bond in PECVD process. Furthermore, the GQDs/MnO_2_ heterostructural electrodes not only can enlarge the operating potential window to 0–1.3 V but also can improve the specific capacitance to 1170 F g^−1^. The extended potential window and improved specific capacitance of GQDs/MnO_2_ heterostructural electrodes can be attributed to the built‐in electric field in heterostructures. To construct ASC, NG was used as anode which is stable in the negative potential window of −1 to 0 V. The 2.3 V aqueous GQDs/MnO_2_‐3//NG ASC exhibited superior electrochemical performance, including high energy density (118 Wh kg^−1^) and high power density (12 351 W kg^−1^). Moreover, this rational design concept of in situ formed heterostructures between GQDs and TMO can be a general strategy for enhancing capacitance and extending voltage window for high energy density aqueous supercapacitors.

## Conflict of Interest

The authors declare no conflict of interest.

## Supporting information

Supplementary
