# Supplementary material for: Heterostructural Graphene Quantum Dot/MnO2 Nanosheets toward High‐Potential Window Electrodes for High‐Performance Supercapacitors
Source: Adv Sci (Weinh). 2018 Mar 6;5(5):1700887. doi: 10.1002/advs.201700887 (PMC5979621; doi:10.1002/advs.201700887)
Supplement: Supplementary file 1 — Supplementary [file ADVS-5-1700887-s001.pdf]

Copyright WILEY-VCH Verlag GmbH & Co. KGaA, 69469 Weinheim, Germany, 2018.

ADVANCED SCIENCE
Open Access

Supporting Information

for *Adv. Sci.,* DOI: 10.1002/advs.201700887

Heterostructural Graphene Quantum Dot/MnO_2 Nanosheets toward High-Potential Window Electrodes for High-Performance Supercapacitors

Henan Jia, Yifei Cai, Jinghuang Lin, Haoyan Liang, Junlei Qi, Jian Cao, Jicai Feng, and WeiDong Fei**

Copyright WILEY-VCH Verlag GmbH & Co. KGaA, 69469 Weinheim, Germany, 2016.

Supporting Information

Heterostructural Graphene Quantum Dots/MnO$_2$ Nanosheets Toward High Potential Window Electrodes for High-Performance Supercapacitors

Henan Jia†, Yifei Cai†, Jinghuang Lin, Haoyan Liang, Junlei Qi, Jian Cao, Jicai Feng, Wei-Dong Fei**

State Key Laboratory of Advanced Welding and Joining, Harbin Institute of Technology,

Harbin 150001, China

*Corresponding authors: Tel. /fax: 86-451-86418146;

†These authors contributed equally.

E-mail: jlqi@hit.edu.cn (J L Qi)

Calculations. The specific capacitance was calculated from CV curves the following equation by integrating discharge current *I*:[1]

$$C = \int IdV / 2\nu mV$$

where *m* is the weight of active materials (g), *v* is the scan rate (mV s^{-1}), and *V* is the sweep potential range of the CV curves (1 V). The power density (*P*, KW Kg^{-1}) and energy density (*E*, W h Kg^{-1}) were calculated from GCD curves,[2]

$$E = \frac{0.5CV^2}{3.6}$$

$$P = \frac{3600E}{t}$$

where *C* is capacitance (F g^{-1}), *V* is the sweep potential range (1V), *t* is the discharge time (s).

Experimental Section

Synthesis of MnO_2 nanosheet arrays on Ni foam: MnO_2 nanosheet arrays on Ni foam were synthesized by a simple hydrothermal method. In a typical procedure, porous 3D Ni foam (3 mm thick, porosity ~95%) was ultrasonically cleaned with 3 M hydrochloric acid and deionized water, to remove the surface nickel oxide layer. $KMnO_4$ (15 mg) and 0.3 M HCl were dissolved in deionized water (40 ml). A piece of cleaned Ni foam was immersed into the solution, and the mixture was transferred to a 50 mL Teflon stainless-steel autoclave. The autoclave was sealed and maintained at 160 °C for 24 h. The autoclave was then allowed to cool to room temperature. The as-prepared materials were washed with deionized water and ethanol several times, and dried under vacuum at 80 °C for 8 h. The mass loading of the MnO_2 nanosheet arrays on the Ni foam was calculated as previously reported.[3,4] Ten MnO_2 samples with a size of 2*5 cm^2 were prepared through a hydrothermal process, and the obtained MnO_2 products were dissolved in 3M HCl for 24 h. After that, the obtained products were washed by water and ethanol for several times. Then, the washed products were dried at 80 ^oC overnight. By weighing these products through a high-precision balance (Denver Instruments, sensitivity: 0.01 mg), the MnO_2 on Ni foam can be estimated to about 1.3 mg cm^{-2} for pure MnO_2. The GQDs/MnO_2 samples were calculated through the same process, and the obtained GQDs/MnO_2 products were dissolved in 3M HCl for 24 h. The active materials on Ni foam can be estimated to about 1.3 mg cm^{-2} to 1.36 mg cm^{-2} for GQD/MnO_2 products, as illustrated in Table S1.

Synthesis of GQDs/MnO_2 heterostructural materials: the as-prepared MnO_2 NAs were placed in the chamber of the PECVD system. the MnO_2 NAs were treated to 350 °C in 15 min in an Ar gas atmosphere (the Ar gas flow was kept at 40 sccm) under a pressure of 200 Pa. Subsequently, a mixture 1:3 Ar/CO_2 gas flow was then introduced into the chamber, to transform the MnO_2 nanosheets to GQDs/MnO_2 heterostructural materials. The total gas

pressure was fixed at 400 Pa. The power of the radio frequency was 200 W, and the deposition time was 0-10 min.

Preparation of GQDs with solution method:[5] 40 mL of H_2O_2 (30%) was added to 5 mL of GO stock solution (1 mg/mL). The mixture solution was heated to 90 ^oC and maintained 12 h with vigorous stirring. After rotary evaporation at 60 ^oC to remove unreacted H_2O_2 and water, ethanol was used as a poor solvent to precipitate and wash the final GQDs product.

Preparation of the H-GQD@MnO$_2$: The H-GQDs@MnO_2 hybrid was prepared by an hydrothermal method. Firstly, the required amount of GQDs product was dissolved in distilled water to give a desired concentration (0.1 mol/L). The homogeneous solution was stirred for 30 min for uniform solution. Then, NaOH solution with a concentration of 0.3 mol/L was slowly added to adjust the pH to 7. After that, MnO_2 nanosheets were immersed into GQDs solutions for 24 h. The as-prepaered materials were washed with distilled water several times, dried at 60 ^oC for 10 h and calcined in Ar atmosphere at 200 ^oC for 2 h.

Materials characterization: The morphology and structure of the samples were characterized by SEM (Helios Nanolab 600i) and TEM (Tecnai G2 F30), respectively. XRD was performed using a D/max 2550 with graphite monochromatized Cu Kα radiation. Raman spectra were collected on a Renishaw inVia Raman Microscope with laser excitation at 532 nm. Surface chemical states were investigated by XPS (Thermo Fisher) with a base pressure of 2×10^{-9} mbar.

Electrochemical Performance Measurements: Electrochemical measurements were conducted in a typical three-electrode cell. The samples were used as working electrodes. Pt foil as the counter electrodes and a saturated calomel electrode (SCE) as reference electrodes in 1 M Na_2SO_4 electrolyte. CV curves and the galvanostatic cycling test of GQD/MnO_2 were carried out on a CHI 760D electrochemical workstation in the potential range of 0-1 V and 0-1.3 V. Electrochemical impedance spectroscopy (EIS) measurements were performed on PARSTAT 4000A electrochemical workstation, over a frequency range from 10^5 to 10^{-2} Hz,

at an amplitude of 5 mV. Further, an asymmetric supercapacitor using GQDs/MnO_2-3 electrodes as positive electrode and nitrogen-doped graphene (NG) as negative electrode were tested in a two-electrode configuration. Nitrogen-doped graphene was prepared through a hydrothermal method.[6] The mass ratio of two electrodes were balanced by the following relationship:

$$m_{+}/m_{-} = (C_{-} \times \Delta E_{-})/(C_{+} \times \Delta E_{+})$$

where *m* (g) is the mass of the electrode materials (anode or cathode), *C* (F/g) is the specific capacitance, and ΔE is the potential window.

Table S1. The mass loading and mass percent of all samples.

	Mass loading (mg cm^{-1})	Mass percent of GQDs (wt.%)	Mass percent of MnO_2 (wt.%)
MnO_2	1.3	0	100
GQDs/MnO_2-1	1.32	1.61	98.39
GQDs/MnO_2-3	1.33	3.22	96.78
GQDs/MnO_2-5	1.35	4.04	95.96
GQDs/MnO_2-10	1.36	4.85	95.15

Table. S2. The energy density and relative performance of various MnO_2-based asymmetric device in references.

electrode materials	electrolyte	specific capacitance ($F\cdot g^{-1}$)	voltage window	Power Density ($W\cdot kg^{-1}$)	Energy Density ($Wh\cdot kg^{-1}$)	Reference
Au-nanoparticles-doped-MnO_2 nanocomposites// NCTs/ANPDM	1 M Na_2SO_4	576 at 1 $A\cdot g^{-1}$	0.8 V	28000	51	7
MnO_2/GO//HPC	1 M Na_2SO_4	84 at 0.1 $A\cdot g^{-1}$	2 V	100	46.7	8
MnO_2-ERGO//CNT–ERGO	1 M Na_2SO_4	69.4 at 0.5 $A\cdot g^{-1}$	1.8 V	453.6	31.8	9
p-BC@MnO_2 //p-BC/N	1 M Na_2SO_4	59.2 at 10$mV\cdot s^{-1}$	2 V	284	32.91	10
Ni nanotube arrays@MnO_2// NiNTAs@Fe_2O_3 nanoneedle	1 M Na_2SO_4	95.9 at 10$mV\cdot s^{-1}$	1.6 V	3197.7	34.1	11
electrodeposited MnO_2//activated carbon	PVA/ Na_2SO_4 gel	100 at 10$mV\cdot s^{-1}$	1.7 V	6227	40.2	12
$NiCo_2O_4$@MnO_2// activated carbon	1 M KOH	83.3 at 0.5 $A\cdot g^{-1}$	1.8 V	187.5	37.5	13
MnO_2 nanoflowers// Bi_2O_3/CNF paper	1 M Na_2SO_4	25.1 at 3 $mA\cdot cm^{-2}$	1.8 V	352.6	11.3	14
Layered V_2O_5/PEDOT/MnO_2 nanosheets//AC	1 M Na_2SO_4	48.2 at 0.5 $mV\cdot s^{-1}$	1.8 V	2200	21.7	15

Ni-Mn-O Nanoprism Arrays	1 M LiCl	242 $mF\cdot cm^{-2}$ at 0.5 $mA\cdot cm^{-2}$	2.4 V	4.72·mWh cm^{-3}	61.2mW cm^{-3}	16
MnO_2 nanorodes (MNR)-rGO// VNW-rGO	1 M LiTFSI	13 at 0.5 $A\cdot g^{-1}$	2 V	456	6	17
$Na_{0.5}MnO_2$// Fe_3O_4@C	1 M Na_2SO_4	86.3 at 1 $A\cdot g^{-1}$	2.6 V	647	81	18
GQDs/MnO_2 nanosheets//nitrogen -doped graphene	1 M Na_2SO_4	**160.6 at 5 $mV\cdot s^{-1}$**	**2.3 V**	**923.3**	**118**	**This work**

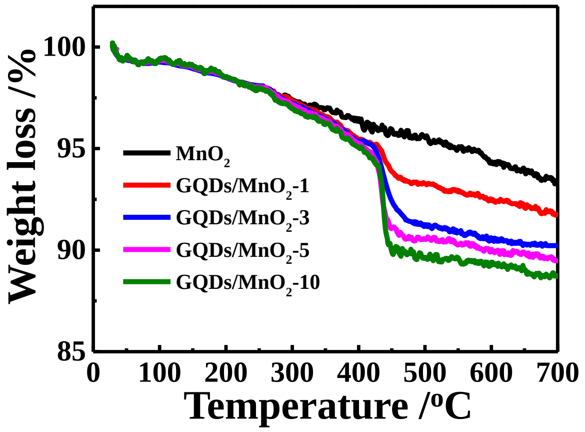

Figure S1. TGA curves of MnO_2, GQDs/MnO_2-1, GQDs/MnO_2-3, GQDs/MnO_2-5 and GQDs/MnO_2-10.

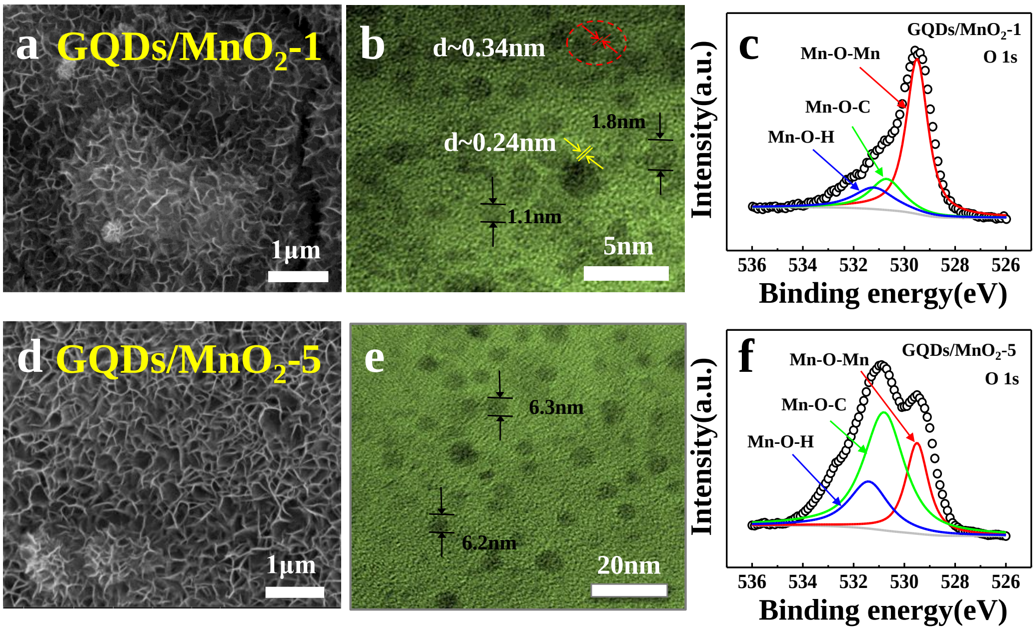

Figure S2. SEM images of a) GQDs/MnO_2-1 and d) GQDs/MnO_2-5. TEM images of b) GQDs/MnO_2-1 and e) GQDs/MnO_2-5. High-resolution O 1s XPS spectra for c) GQDs/MnO_2-1 and f) GQDs/MnO_2-5.

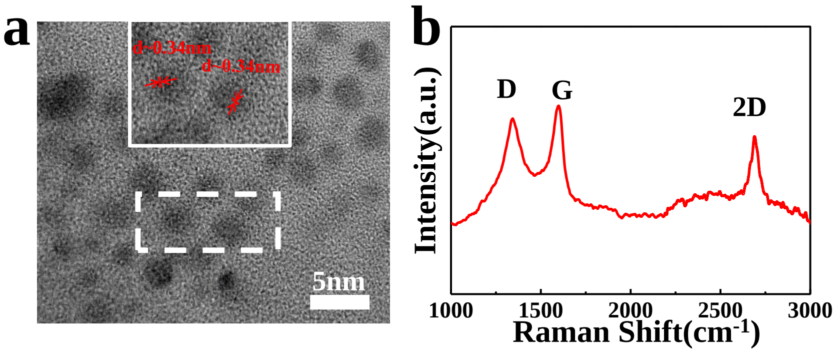

Figure S3. a) the HRTEM image and b) Raman spectrum of GQDs/MnO_2-3.

Carbon-based quantum dots consisting of graphene quantum dots (GQDs) and carbon quantum dots (CQDs) are a new class of carbon nanomaterials,[19] but GQDs and CQDs are structural distinct.[20] CQDs are quasi-spherical nanoparticles usually <100 nm in diameter, and some of CQDs are always with hollow structure.[21] They can be always amorphous with nanometre-sized sp^2 hybridized graphitic cores.[22] In contrast, GQDs resemble the crystalline structure of single or few-layered graphene, exhibits unique properties.[23, 24] The lateral dimension of GQDs is always about a few nanometers.[25] Compared with conventional CQDs, GQDs are generally smaller and of higher crystallinity.[20] And GQDs have larger specific surface areas and more accessible edges, which results in an influence of capacitance. In our case, HRTEM images of the GQDs/MnO_2-3 shows that many dots are uniformly distributing in the field of vision, as shown in Figure 3a. The sizes of GQDs in GQDs/MnO_2-3 are approximately 2-3 nm, and the lattice spacing of 0.34 nm is excellently indexed to the (002) spacing of graphitic carbon, which implies small sizes and high crystallinity, and this agrees with the previous report about GQDs.[20] This result indicates that carbon-based quantum dots with higher crystallinity should be GQDs.

In order to illustrate the unique structure of GQDs, the Raman spectrum of GQDs/MnO_2 was measurement, as shown in Figure 3b. The Raman spectrum also contains characteristic D and G bands of carbon at approximately 1350 and 1580 cm^{-1}. The G band is stronger than D band, and a sharp G band indicates better crystallinity of the GQDs. However, the edges are always

seen as defects, which cause the D band.[26] And an additional peak located in 2695 cm^{-1}, corresponding to 2D band, can also be observed, which agrees with previous reports.[27-29] This weak 2D band without a typical graphite shoulder and shows low I_{2D}/I_G ratio (0.5) implying the few-layered feature of GQDs in GQDs/MnO_2,[30] which further prove the formation of GQDs.[23]

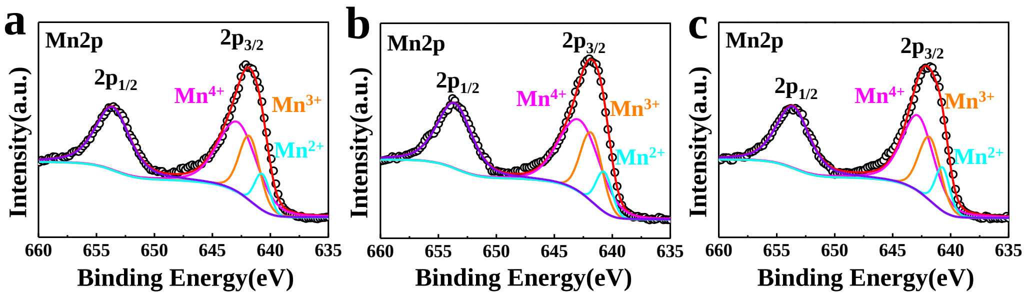

Figure S4. High-resolution Mn 2p XPS spectra for a) MnO_2, b) GQDs/MnO_2-3, and c) GQDs/MnO_2-10.

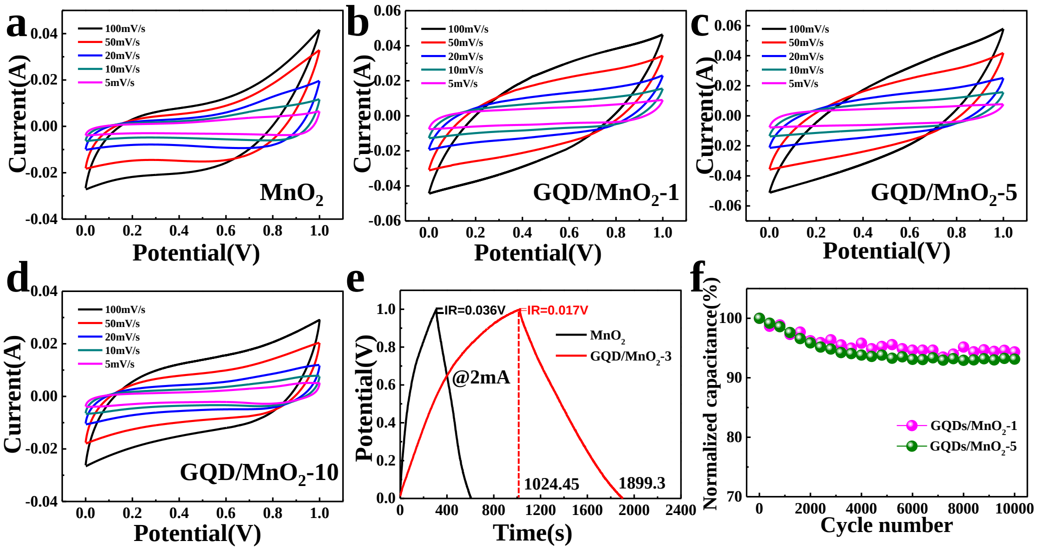

Figure S5. CV curves at different scan rates (5-100 mV s^{-1}) for a) MnO_2, b) GQDs/MnO_2-1, c) GQDs/MnO_2-5 and d) GQDs/MnO_2-10. e) GCD curves of MnO_2 and GQDs/MnO_2-3 at current density of 1 A g^{-1}. f) cycling stability tests over 10000 cycles for GQDs/MnO_2-1 and GQDs/MnO_2-5.

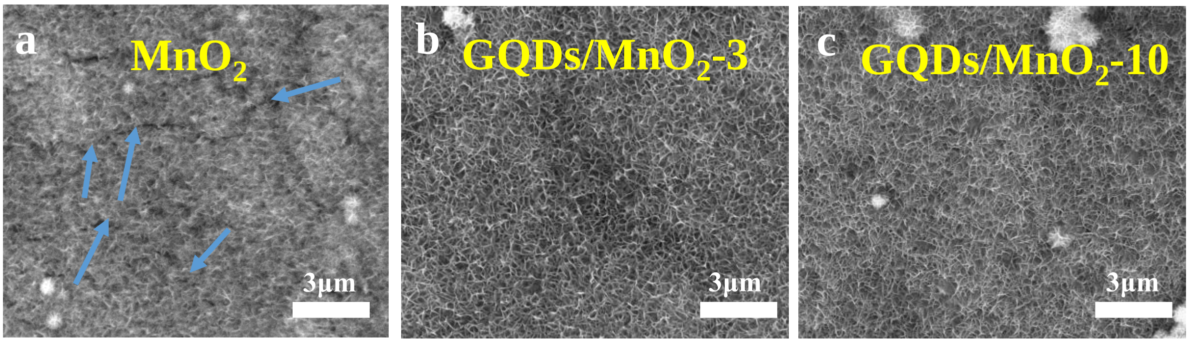

Figure S6. SEM images of a) MnO_2 nanosheet arrays, b) GQDs/MnO_2-3 and c) GQDs/MnO_2-10 after cycling tests.

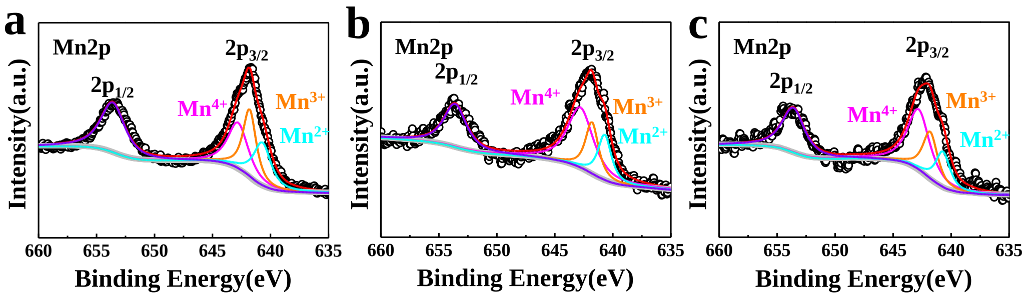

Figure S7. High-resolution Mn 2p XPS spectra after cycling test for a) MnO_2, b) GQDs/MnO_2-3, and c) GQDs/MnO_2-10.

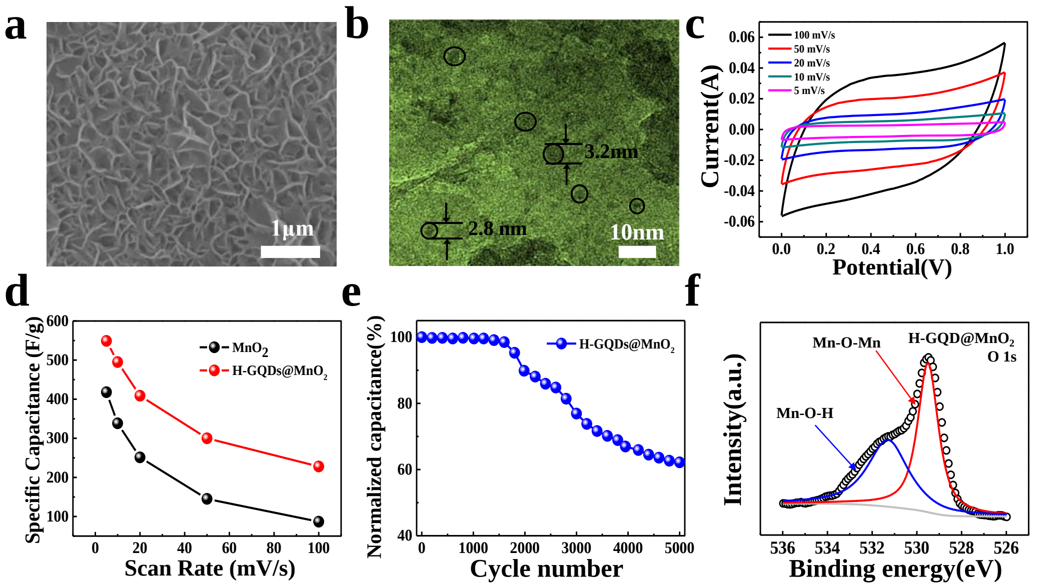

Figure S8. a) SEM image and b) TEM image of H-GQDs@MnO_2. c) CV curves of H-GQDs@MnO_2 at different scan rates (5-100 mV s^{-1}). d) Specific capacitances of H-GQDs@MnO_2 as a function of the scan rate. e) cycling stability tests over 5000 cycles. f) High-resolution O 1s XPS spectra for H-GQDs@MnO_2.

Figure S8a shows the SEM image of H-GQDs@MnO_2. It can be seen that the nanosheet structure is remained in H-GQDs@MnO_2. Figure S8b exhibits the HRTEM image of H-GQDs@MnO_2, and it illustrates the size of GQDs in H-GQDs@MnO_2 is about 2-3 nm. The CV curves of H-GQDs@MnO_2 with various sweep rates ranging from 5 to 100 mV/s is shown in Figure S8c. Compared with that of pure MnO_2 nanosheet arrays, CV curve of H-GQDs@MnO_2 is relatively rectangular in shape and exhibits a larger area, which higher capacitance than that of pure MnO_2 nanosheet arrays. The specific capacitance of H-GQDs@MnO_2 can reach 550 F/g at a scan rate of 5 mV/s, which means that GQDs play an important role to improve can improve the conductivity and electrochemical performances. However, there is a limited improvement of specific capacitance and a poor cycling stability (62.2% retention after 10000 cycles, as shown in Figure S3e), which can be attributed to the inexistence of Mn-O-C bond through impregnation method (as shown in Figure S8f).

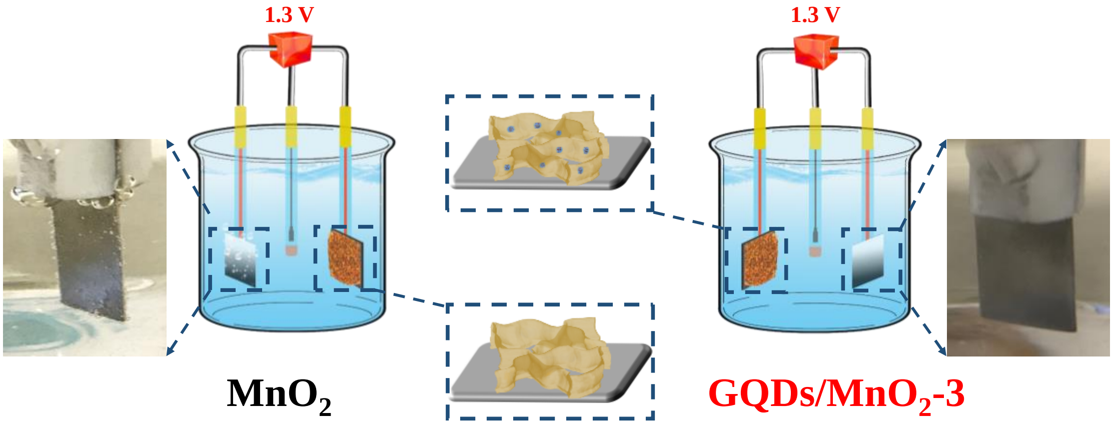

Figure S9. the digital pictures of electrolytic cell in different measurement conditions for MnO_2 and GQDs/MnO_2-3.

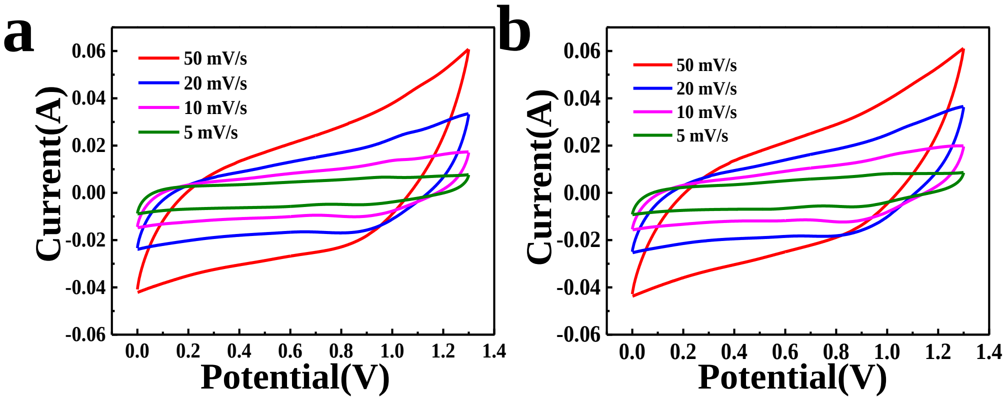

Figure S10. a) CV curves of GQDs/MnO_2-1 at different scan rates (5-50 mV s^{-1}). b) CV curves of GQDs/MnO_2-10 at different scan rates (5-50 mV s^{-1}).

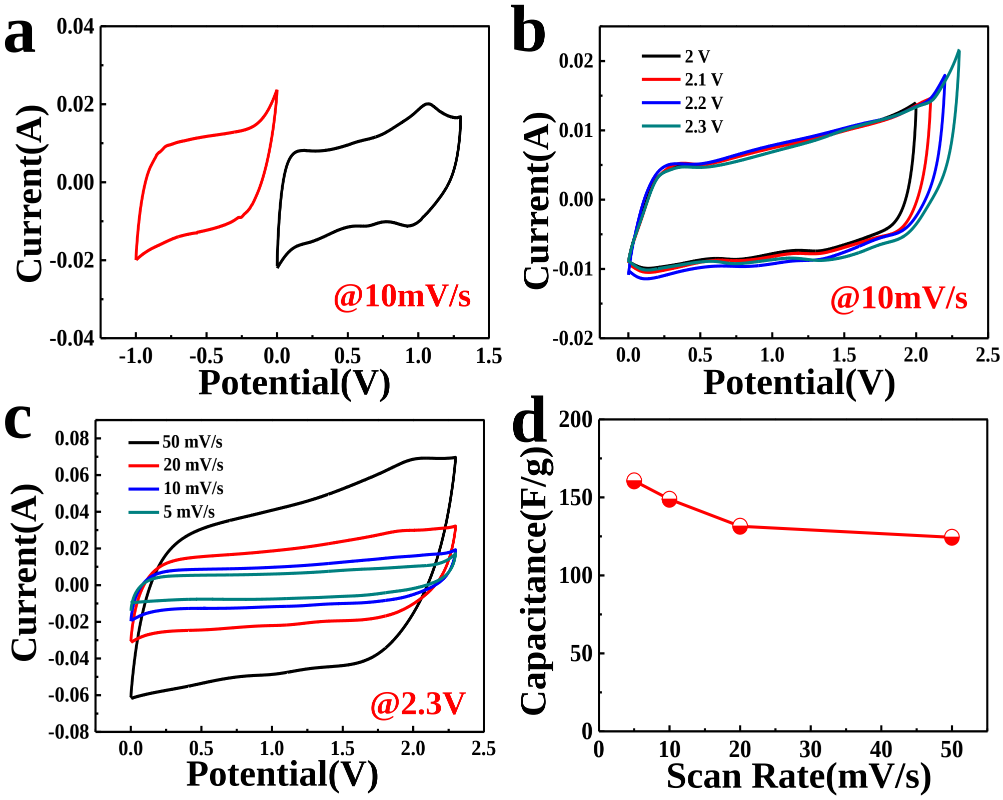

Figure S11. a) CV curves of the typical GQDs/MnO_2-3 electrodes and NG at the scan rate of 10 mV/s. b) CV curves of GQDs/MnO_2-3//NG ASC in different voltage window of 0-2 V, 0-2.1 V, 0-2.2 V and 0-2.3 V at 10 mV/s. c) CV curves of GQDs/MnO_2-3//NG ASC in different scan rate. d) The specific capacitances of the GQDs/MnO_2-3//NG ASC as a function of scan rate.

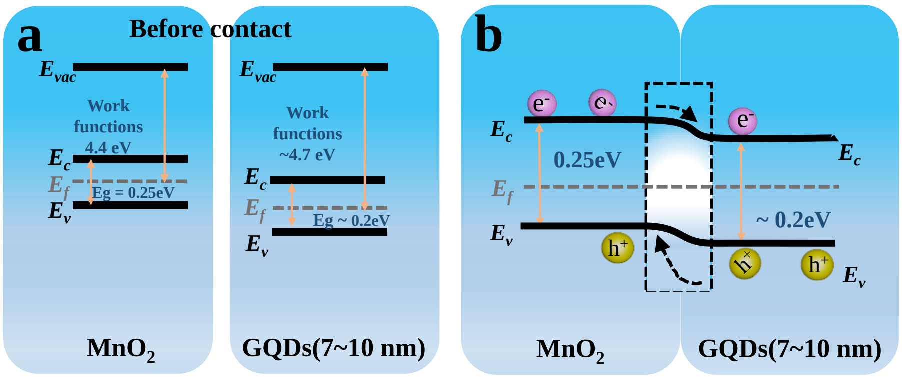

Figure S12. a) Energy diagram of MnO_2 and GQDs (7~10 nm) before contact. (h) Energy diagram of the interface between MnO_2 and GQDs (7~10 nm) after the formation of a heterojunction.

Reference:

(1) E. Eustache, C. Douard, R. Retoux, C. Lethien, T. Brousse, *Adv. Energy Mater.* **2015**, *5*, 1500680.

(2) Y. Huang, Y. Li, Z. Hu, G. Wei, J. Guo, J. Liu, *J. Mater. Chem. A* **2013**, *1*, 9809.

(3) Y. Sun, W. Zhang, D. Li, L. Gao, C. Hou, Y. Zhang, Y. Liu, *Electrochim. Acta* **2015**, *178*, 823.

(4) T. Zhu, S. J. Zheng, Y. G. Chen, J. Luo, H. B. Guo, Y. E. Chen, *J. Mater. Sci.* **2014**, *49*, 6118.

(5) F. Jiang, D. Chen, R. Li, Y. Wang, G. Zhang, S. Li, J. Zheng, N. Huang, Y. Gu, C. Wang, C. Shu, *Nanoscale* **2013**, *5*, 1137.

(6) Y. Wang, Z. D. Wei, Y. Nie, Y. Zhang, *J. Mater. Chem. A* **2017**, *5*, 1442.

(7) Q. Lv, S. Wang, H. Sun, J. Luo, J. Xiao, F. Xiao, S. Wang, *Nano Lett.* **2015**, 16, 40.

(8) Y. Zhao, W. Ran, J. He, Y. Huang, Z. Liu, W. Liu, Y. Tang, L. Zhang, D. Gao, F. Gao, *Small* **2015**, 11, 1310.

(9) Z. Zhang, F. Xiao, L. Qian, J. Xiao, S. Wang, Y. Liu, *Adv. Energy Mater.* **2014**, 4, 1400064.

(10) L. F. Chen, Z. H. Huang, H. W. Liang, Q. F. Guan, S. H. Yu, *Adv. Mater.* **2013**, 25, 4746-4752.

(11) Y. Li, J. Xu, T. Feng, Q. Yao, J. Xie, H. Xia, *Adv. Funct. Mater.* **2017**, 27, 1606728.

(12) N. Liu, Y. Su, Z. Wang, Z. Wang, J. Xia, Y. Chen, Z. Zhao, Q. Li, F. Geng, *ACS Nano* **2017**, 11, 7879.

(13) Y. Zhang, B. Wang, F. Liu, J. Cheng, X. Zhang, L. Zhang, *Nano Energy* **2016**, 27, 627.

(14) H. Xu, X. Hu, H. Yang, Y. Sun, C. Hu, Y. Huang, *Adv. Energy Mater.* **2015**, 5, 1401882.

(15) C. X. Guo, G. Yilmaz, S. Chen, S. Chen, X. Lu, *Nano Energy* **2015**, 12, 76.

(16) W. Zuo, C. Xie, P. Xu, Y. Li, J. Liu, *Adv. Mater.* **2017**, 27, 1703463.

(17) S. D. Perera, M. Rudolph, R. G. Mariano, N. Nijem, J. P. Ferraris, Y. J. Chabal, K. J. Balkus, *Nano Energy* **2013**, *2*, 966.

(18) N. Jabeen, A. Hussain, Q. Xia, S. Sun, J. Zhu, H. Xia, *Adv. Mater.* **2017**, *29*, 1700804.

(19) Y. Wang, A. Hu, *J. Mater. Chem. C* **2014**, *2*, 6921.

(20) K. D. Wegner, S. Lindén, Z. Jin, T. L. Jennings, R. E. Khoulati, P. M. P. van Bergen En Henegouwen, N. Hildebrandt, *Small* **2014**, *10*, 734.

(21) Y. Zhai, Z. Zhu, C. Zhu, J. Ren, E. Wang, S. Dong, *J. Mater. Chem. B* **2014**, *2*, 6995.

(22) W. Kwon, S. Rhee, *Chem. Commun.* **2012**, *48*, 5256.

(23) D. Liu, X. Chen, Y. Hu, T. Sun, Z. Song, *Nat. Commun.* **2018**, *9*, 193.

(24) H. Zhao, Y. Chang, M. Liu, S. Gao, H. Yu, X. Quan, *Chem. Commun.* **2013**, *49*, 234.

(25) R. Liu, D. Wu, X. Feng, K. Müllen, *J. Am. Chem. Soc.* **2011**, *133*, 15221.

(26) J. Kim, J. S. Suh, *ACS Nano* **2014**, *8*, 4190.

(27) Z. H. Ni, T. Yu, Y. H. Lu, Y. Y. Wang, Y. P. Feng, Z. X. Shen, *ACS Nano* **2008**, *2*, 2301.

(28) DI Son, B. W. Kwon, D. H. Park, W. S. Seo, Y. Yi, *Nat. Nanotechnol.* **2012**, *7*, 465.

(29) F. Liu, M. Jang, H. D. Ha, J. Kim, Y. Cho, T. S. Seo, *Adv. Mater.* **2013**, *25*, 3657.

(30) Y. Zhu, S. Murali, W. Cai, X. Li, J. W. Suk, J. R. Potts, R. S. Ruoff, *Adv. Mater.* **2010**, *22*, 3906.
